# Supplementary material for: Enhancing Radiotherapy Sensitivity in Prostate Cancer with Lentinan-Functionalized Selenium Nanoparticles: Mechanistic Insights and Therapeutic Potential
Source: Pharmaceutics. 2024 Sep 21;16(9):1230. doi: 10.3390/pharmaceutics16091230 (PMC11434965; doi:10.3390/pharmaceutics16091230)
Supplement: Supplementary file 1 [file pharmaceutics-16-01230-s001.zip › pharmaceutics-3162943-supplementary.pdf]

Results

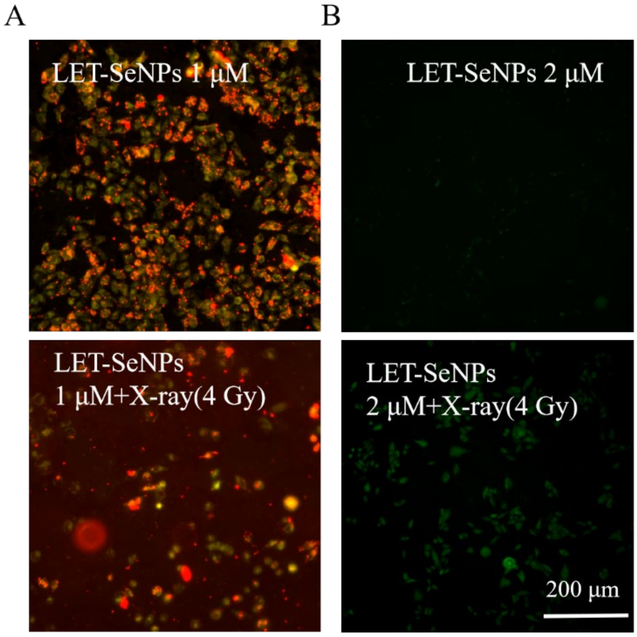

Figure S1 After 2 hours of treatment with LET-SeNPs combined with X-ray radiotherapy (4 Gy), the ROS levels in PC3 cells were measured. (A) JC-1 fluorescence detected by fluorescent microscope after treated with concentration of LET-SeNPs (1 μM) and X-ray (4 Gy). (B) Fluorescence imaging in PC3 cells marked with DCF after treated with concentration of LET-SeNPs (2 μM) and X-ray (4 Gy).

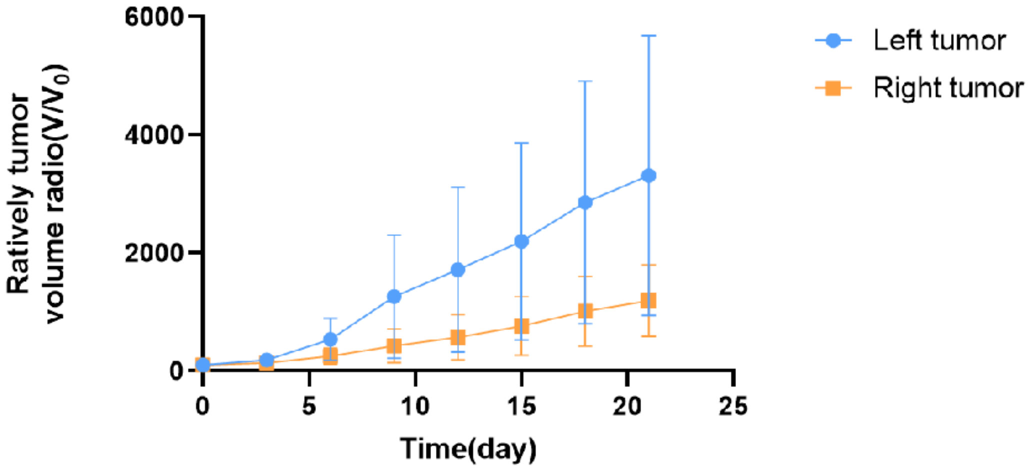

Figure S2 In bilateral tumor bearing model, the tumor volume changed on both sides of the same mouse within 21 days of treatment.
